# Keeping ribosomal DNA intact: a repeating challenge

**DOI:** 10.1007/s10577-018-9594-z

**Published:** 2018-12-17

**Authors:** Daniël O. Warmerdam, Rob M. F. Wolthuis

**Affiliations:** 1 CRISPR Platform, University of Amsterdam, Cancer Center Amsterdam, Amsterdam UMC, Meibergdreef 9, 1105 AZ Amsterdam, the Netherlands; 2 Section of Oncogenetics, Department of Clinical Genetics, Vrije Universiteit Amsterdam, Cancer Center Amsterdam, Amsterdam UMC, de Boelelaan 1117, 1081 HV Amsterdam, the Netherlands

**Keywords:** Repetitive DNA, Ribosomal DNA, DNA repair, DNA repeat integrity, Bloom Syndrome, Ataxia-Telangiectasia, Genomic instability, CRISPR

## Abstract

More than half of the human genome consists of repetitive sequences, with the ribosomal DNA (rDNA) representing two of the largest repeats. Repetitive rDNA sequences may form a threat to genomic integrity and cellular homeostasis due to the challenging aspects of their transcription, replication, and repair. Predisposition to cancer, premature aging, and neurological impairment in ataxia-telangiectasia and Bloom syndrome, for instance, coincide with increased cellular rDNA repeat instability. However, the mechanisms by which rDNA instability contributes to these hereditary syndromes and tumorigenesis remain unknown. Here, we review how cells govern rDNA stability and how rDNA break repair influences expansion and contraction of repeat length, a process likely associated with human disease. Recent advancements in CRISPR-based genome engineering may help to explain how cells keep their rDNA intact in the near future.

## Introduction

Two aspects of the ribosomal DNA (rDNA) are very remarkable: first, it contains hundreds of repeated genes, and secondly, it forms the most heavily transcribed region in the human genome. The ribosomal RNA (rRNA) transcribed from the approximately 600 rDNA repeats forms the most abundant fraction of RNA found in eukaryotic cells (McStay 2016). Together with about ~ 80 proteins, representing 10% of cellular protein levels, the rRNAs are built into ribosomes, the macromolecular machines through which messenger RNAs (mRNAs) are guided during protein synthesis (Boisvert et al. 2007).

Ribosome biogenesis, the process of ribosome assembly, involves the coordinated function of more than 200 proteins and occurs both in the cytoplasm and in the nucleolus (Thomson et al. 2013). This is also a major energy consuming process which is tightly controlled by the availability of nutrients and growth factors (Boulon et al. 2010). During favorable conditions for cellular growth and division, rRNA production is high, while in response to stress, such as nutrient starvation or DNA damage, rDNA transcription is efficiently repressed (Boulon et al. 2010). Thus, cells have evolved an intricate feedback network to balance rDNA production to their cellular environment and changing growth conditions.

Repetitive regions of DNA, by their very nature, are prone to DNA recombination events (Stankiewicz and Lupski 2002). Recombination events can result in a reduction of the repeat copy numbers or in DNA mutations (Carvalho and Lupski 2016). To prevent these, multiple advanced DNA repair mechanisms are in place to maintain rDNA repeat integrity (Larsen and Stucki 2016; van Sluis and McStay 2017). The importance of such mechanisms is underscored by the diseases associated with deficiencies in DNA caretaker genes like CSA and CSB (Cockayne syndrome), BLM (Bloom syndrome), WRN (Werner syndrome), and ATM (ataxia-telangiectasia). Cells derived from patients suffering from these conditions also display features of increased rDNA instability, accompanied by poor ribosome biogenesis and, potentially, defective protein synthesis (Killen et al. 2009; Stults et al. 2008). This suggests that rDNA instability may contribute to some of the clinical presentations of these diseases (Killen et al. 2009; Christians and Hanawalt 1994). In this review, we will focus on the role of rDNA instability in human disease and discuss the mechanisms involved in maintaining rDNA repeat integrity. We draw special attention to the pathways involved in the repair of DNA double-stranded breaks (DSBs) in the highly repetitive rDNA, as these breaks form an immediate threat to the transcription and stability of rDNA and therefore can attribute to human diseases including neurodegeneration and cancer.

## Ribosomal DNA repeats

The rDNA repeats reside in the nucleolus, a membrane-less sub-nuclear compartment which assembles around chromosomal nucleolar organizing regions consisting of clusters of rRNA gene repeats (McStay 2016). Nucleoli are formed through liquid-liquid phase separation, which keeps them interlinked with the rest of the nucleus and enables them to rapidly disassemble and reassemble as cellular conditions change (Feric et al. 2016; Hult et al. 2017; Falahati and Wieschaus 2017; Mitrea and Kriwacki 2016; Tang 2017). Heterochromatic rDNA is found in close proximity to the nucleolus while transcriptional active rDNA resides within, at the boundary of the fibrillar centers and the dense fibrillar compartments (Pontvianne et al. 2013; Zentner et al. 2011; Nemeth et al. 2008).

**Fig. 1:**
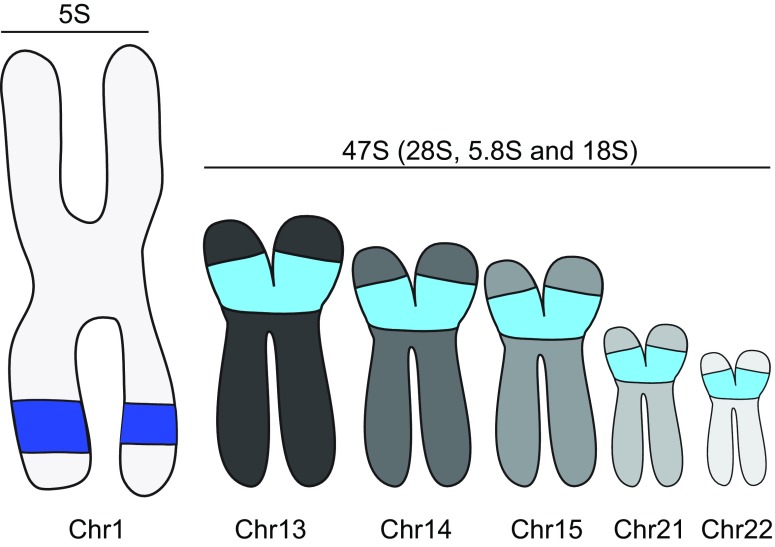
Graphical illustration of the chromosome locations of the 47S and 5S rDNA repeats. The 47S repeats are distributed over five different chromosomes, whereas the 5S repeats are all located on chromosome 1

The human rDNA encompasses several genomic loci and is mostly organized in head-to-tail tandem repeats (Worton et al. 1988). The 5S rDNA region on chromosome 1 encodes the 5S rRNA gene repeats and contains structural intergenic spacer regions. The genes encoding the 18S, 5.8S, and 28S (47S) rRNAs are distributed over chromosomes 13, 14, 15, 21, and 22 (Fig. 1) and are also characterized by intergenic spacer regions (Gibbons et al. 2014). While the copy numbers of rDNA can vary both across and within species, the 5S and 47S loci each contains approximately 300 repeats in human cells. Interestingly, the number of repeats of the 5S and 47S loci evolved in a correlated fashion. Although it is unclear how its numbers are conserved (Gibbons et al. 2015), this indicates the existence of robust cellular mechanisms controlling rDNA repeat integrity and underscores the importance of maintaining repeat stability for cellular homeostasis.

## Ribosomal DNA repeat instability in human disease

In general, DNA repeat instability can cause human disease as illustrated by the observation of pathogenic repeat expansions in many neurological disorders like amyotrophic lateral sclerosis (ALS), frontotemporal dementia (FTD), and Huntington’s disease (Hannan 2018). Considering their susceptibility for genomic instability, it is not surprising that also rDNA repeats are associated with human disorders. Persistent rDNA damage signaling and structural rearrangements following erroneous repair, as well as consequential transcriptional alterations and ribosome dysfunction, may trigger disease (Diesch et al. 2014). Disruption of genes that function directly in ribosome biogenesis, so-called ribosomopathies, is associated with bone marrow failure and skeletal malformations as seen in Diamond-Blackfan Anemia, Schwachman-Diamond Syndrome, and Dyskeratosis Congenita (Narla and Ebert 2010). In addition, several DNA caretaker genes, traditionally associated with cancer predisposition and premature aging syndromes, have been implicated in maintaining ribosome function. Loss of functional BLM, WRN, and ATM leads to rDNA repeat instability. Moreover, TCOF1, which is mutated in Treacher Collins syndrome and operates in close association with DNA damage response protein NBS1 (mutated in Nijmegen breakage syndrome), is involved in the regulation of rDNA transcription (Killen et al. 2009, Caburet et al. 2005, Ciccia et al. 2014, Hannan et al. 2013a, b). Cohesinopathies, including Cornelia de Lange and Roberts Syndrome, are also associated with nucleolar alterations (Xu et al. 2014; Gard et al. 2009). Altogether, defects in genomic caretakers correlate with lower expression levels of rRNA and/or nucleolar dysfunction which may impact ribosome assembly (Diesch et al. 2014). However, it remains an important question whether clinical outcomes are a direct consequence of rDNA instability and altered ribosome biogenesis, or are associated predominantly with deficiencies in overall genomic DNA maintenance.

### Neurodegeneration

A hexanucleotide repeat expansion in the C9orf72 gene causes ALS and FTD (Haeusler et al. 2014). The repeat extension results in (i) DNA-RNA hybrid structures that are known as R-loops, composed of a displaced single-stranded DNA hybridized with the nascent transcript (Santos-Pereira and Aguilera 2015), (ii) harmfully high levels of repetitive C9orf72 RNA, and (iii) toxic dipeptide repeat proteins (Balendra and Isaacs 2018). These three implications clearly link repeat alterations to neurodegenerative diseases. Cells of patients with ataxia-telangiectasia and Bloom syndrome contain up to 100 more rDNA repeats than normal cells and are characterized not only by defective DNA repair but also by neurological impairments (Killen et al. 2009; Stults et al. 2008; Hallgren et al. 2014; Toro et al. 2018; McKinnon 2009). Conversely, altered rDNA repeat numbers have been identified in cells derived from patients diagnosed with these and other neurological disorders (Hallgren et al. 2014; Chestkov et al. 2018). Combined, these observations suggest that repair-dependent rDNA repeat alterations may contribute to the observed neurological disease symptoms in ataxia-telangiectasia and Bloom patients (Hallgren et al. 2014). Several observations indicate how repeat instability may contribute to neurodegeneration. Neurodegeneration is induced by cellular stress, caused by DNA damage and/or toxic levels of RNA and misfolded proteins. Potential factors that could influence rDNA-mediated neurodegeneration are as follows: (i) increased rDNA transcription, leading to rDNA damage and/or toxic levels of rRNA transcripts, (ii) changes in ribosome biogenesis leading to altered protein production, either through toxic high levels or reduced levels that lead to protein deficiency or insoluble aggregates (Slomnicki et al. 2016; Hetman and Slomnicki 2018), and (iii) increased genome instability due to rDNA recombination-mediated structural chromosomal rearrangements. These threats are not necessarily mutually exclusive so rDNA repeat instability might lead to neurodegeneration through cumulative mechanisms.

### Cancer

Analysis of a subset of human lung and colon cancers indicated that half of these solid tumors contain rDNA rearrangements, with one-third of these cancers revealing repeat expansions (Stults et al. 2009). As indicated above, alterations in rDNA copy numbers influence the biology of ribosome formation and homeostasis (Orsolic et al. 2016), thereby affecting protein synthesis rates, quality control, and protein homeostasis. Tumors often appear addicted to high levels of ribosome activity because of their reliance on increased protein production (White and Vijg 2016). Nevertheless, it remains unclear whether rDNA instability itself may act as a driver of oncogenic transformation, although some studies suggest this (Tsoi et al. 2017). A better understanding of the contractile behavior of ribosomal DNA repeats in normal and cancer cells is needed to clarify the processes that contribute to enhanced ribosome activity and thereby provide a mechanism-based rationale for the use of therapeutic drugs that inhibit ribosome biogenesis, either in the prevention or treatment of cancer cells.

## Maintaining ribosomal DNA repeats and RNA transcripts

In eukaryotes, the copy number of rDNA repeats is higher than required to maintain rRNA synthesis. Many copies of rDNA genes are transcriptionally silenced via histone modification and/or methylation (Birch and Zomerdijk 2008). The reason for this wide-spread redundancy is unclear; however, it is possible that certain specific cell types or developmental stages require increased levels of protein synthesis (Russell and Zomerdijk 2005). The inactive fraction of rDNA is organized into a tightly packed, heterochromatic state that may be crucial for the structure of the nucleolus and regulation of rDNA transcription (Tsekrekou et al. 2017). Importantly, the packed state of rDNA heterochromatin could actually determine genomic instability as it is, on the one hand, less accessible to damaging metabolic by-products and, on the other hand, to proteins involved in recombination pathways. Moreover, it has been suggested that rDNA heterochromatin has a DNA damage signaling role: deletion of inactive rDNA copies may trigger a DNA damage response (DDR) followed by apoptosis or senescence (Kobayashi 2008; Paredes and Maggert 2009). The loss of rDNA repeats, even if they are not actively transcribed (i.e., the redundant copies), has also been shown to sensitize cells to mutagen-induced DNA damage (Xu et al. 2017a, b). Recent evidence also suggests that reduction of ribosomal DNA repeats is a trait of human aging (Ganley and Kobayashi 2014; Tiku and Antebi 2018). On the other hand, the contraction of rDNA copies has been proposed as a mechanism that overcomes replication stress conditions, acting as an adaptive cellular response, making it easier for cells with reduced rDNA repeats to complete DNA replication and continue cell cycle progression. Combined, these observations indicate that maintaining appropriate activity and numbers of rDNA repeats is highly controlled as loss of rDNA stability can be detrimental to cells and that replication and transcription are key aspects in the regulation of rDNA.

### Replication of ribosomal DNA

Interestingly, not all rDNA repeats are replicated at the same time during S-phase. Actively transcribed repeats are replicated right after the initiation of DNA replication, whereas the silent repeats are replicated from mid to late S-phase (Dimitrova 2011; Schlesinger et al. 2009). In rDNA that is associated with the nucleolus, actively transcribed rDNA relocates transiently to the periphery of the nucleolar body for replication, possibly to avoid collisions between replication and transcription machineries (Kobayashi 2008). In yeast, the non-transcribed spacer region of a rDNA repeat serves as a binding domain for the Fork blocking protein 1 (Fob1) and a localized increase of this protein is associated with a stronger replication block, to prevent clashes between rDNA transcription and the DNA replication machinery (Kobayashi 2003; Castan et al. 2017). Notably, Fob1 oligomerization has been documented to bring rDNA spacer regions together in a process called “chromosome kissing,” which may increase recombination events (Choudhury et al. 2015; Labib and Hodgson 2007). Surprisingly, an integral protein of the RNA-interference pathway, Dcr1, was shown to be essential for transcription termination at sites of rDNA replication stress in fission yeast, thereby actually preventing recombination events (Sinkkonen et al. 2010; Gadaleta and Noguchi 2017; Castel et al. 2014).

Most eukaryotic cells have evolved a gene amplification system that serves to maintain high rDNA repeat copy numbers and to compensate for any loss in repeats. When repetitive rDNA is reduced in yeast, replication in the rDNA is stalled at the replication fork blocking (RFB) site. Uncoupling of the stalled replication forks results into DSBs that enhance recombination and subsequent repeat expansion (Kobayashi et al. 1998; Akamatsu and Kobayashi 2015). The histone deacetylase Sir2 negatively regulates the RFB and thereby limits excessive recombination in order to maintain a balanced number of rDNA repeats (Kobayashi et al. 2004; Gadaleta and Noguchi 2017). In mammalian cells, an RFB downstream of the 47S pre-rRNA gene is imposed by the RNA Polymerase I (PolI) transcription terminator complex involving transcription termination factor 1 and the replisome protein TIMELESS (Akamatsu and Kobayashi 2015). Regulation of protein-mediated RFBs therefore seems to be crucial in forcing rDNA repeat extensions (Beuzer et al. 2014). Accordingly, it has been suggested that loss of rDNA repeats is a sign of previous events of replication stress (Salim et al. 2017). The histone chaperone Asf1 is known to prevent rDNA repeat expansions in yeast (Houseley and Tollervey 2011), but it remains unclear which counteracting mechanisms control the number of repeats in human cells. Loss of SMC5, BRCA1, and BRCA2 and a number of other genes important in maintaining genome integrity after DNA damage result in rDNA repeat instability (Caburet et al. 2005; Warmerdam et al. 2016; Killen et al. 2009; Thompson and Schild 2002). This implies that also in human cells, rDNA copy numbers are controlled through DNA replication and recombination-associated mechanisms. Maintaining a stable number of repeats is however not the only way in which cells can adjust the appropriate production of rRNA and thereby maintain proficient ribosome synthesis to support translation capacity. Transcription of the rDNA can also be increased by epigenetically reactivating silenced repeats or through increased PolI activity as discussed in the following paragraph.

### Transcription of ribosomal DNA

The rDNA is one of the most actively transcribed regions in the genome. In mammalian cells, initiation of rDNA transcription is controlled by the cell cycle, showing the highest levels of rDNA expression during S and G2-phases, followed by silencing during mitosis, and a gradual re-activation in G1 phase (Huang et al. 2016). During interphase, nucleoli form around the rDNA repeats encompassing the 47S loci, which are then transcribed by PolI (McStay and Grummt 2008). The 47S polycistronic pre-rRNA is spliced into the 28S, 5.8S, and 18S transcripts. The 5S repeat, however, is transcribed outside of the nucleolus by RNA Polymerase III, producing the 5S transcript. Combined, these four transcripts are part of the 60S (28S, 5.8S, and 5S) and 40S (18S) ribosomal subunits.

High rates of rDNA transcription increase the likelihood of R-loop formation (Santos-Pereira and Aguilera 2015). Although these structures have also been implied in supporting rDNA integrity, by facilitating homologous recombination (HR) (Hall et al. 2017), the processing of R-loops may form obstacles for the DNA replication machinery and result in rDNA breaks. The notion that R-loops comprise an endogenous source of genome instability (Amon and Koshland 2016) is supported by the evolution of protective mechanisms which limit, prevent, or resolve R-loops. These include roles for RNAse enzymes that degrade the RNA strand, helicases which unwind the DNA-RNA hybrids or prevent their formation, and topoisomerases mediating dissolution of blocked DNA during replication and transcription (Aguilera and Gomez-Gonzalez 2008; Hamperl and Cimprich 2014). In human disease, R-loop-mediated rDNA damage has been linked to Borjeson-Forssman-Lehmann syndrome (PHF6), Friedreich ataxia (FXN), amyotrophic lateral sclerosis type 4 (SETX), and Fragile X syndrome (FMR1), among others. Also in cancer, mutations in genes involved in suppression of R-loops have been identified, including BRCA1, PHF6, FIP1L1, BREI, and SRSF1 (Santos-Pereira and Aguilera 2015). While it remains unclear whether oncogenic transformation can solely be attributed to R-loop accumulation, the idea that R-loops are a significant source of DNA damage in cancer cells and other disorders has solid support (Lindstrom et al. 2018).

Interestingly, plant homeodomain finger protein 6 (PHF6) was shown to suppress R-loops and subsequent breaks in the rDNA (Wang et al. 2013), identifying it as a negative regulator of rDNA transcription. PHF6 is a highly conserved gene in vertebrates, likely to be essential for development, although no knockout mice have been reported (Crawford et al. 2006). PHF6 contains two PHD domains that are normally associated with chromatin regulation and gene expression. It also interacts with the nucleosome remodeling deacetylase (NuRD) complex, which mediates chromatin assembly. Thereby NuRD supports transcription, cell cycle progression, and genome stability (Todd and Picketts 2012). PHF6 localizes both inside the nucleus and nucleolus (Todd et al. 2016). PHF6 contains putative DDR-dependent phosphorylation sites that suggest that it is regulated in response to genotoxic stress (Todd et al. 2015; Matsuoka et al. 2007). Although we do not yet understand the cellular roles of PHF6, these data suggest an important function for PHF6 in the regulation of rDNA transcriptional output. Mutations in PHF6 are associated with Borjeson-Forssman-Lehmann syndrome and are also implicated in the development of cancer (Van Vlierberghe et al. 2010; Lower et al. 2002), supporting the notion that deregulation of rDNA transcription and rDNA R-loop resolution can result in disease.

Approximately one-third of the rDNA repeats are epigenetically silenced by the nucleolar chromatin remodeling complex (NoRC), comprising Tip5 and Snf2H. NoRC loss impairs rDNA silencing, resulting in an upregulation of rDNA transcription (Guetg et al. 2010). Maintaining appropriate levels of rDNA transcription is crucial for cellular homeostasis since its deregulation can lead to either cell death or oncogenic transformation (Russell and Zomerdijk 2005; Diesch et al. 2014). Indeed, apart from cancer, upregulation of rDNA transcription is also associated with cardiovascular disease (Hariharan and Sussman 2014) and downregulation of rDNA expression is a common cellular feature of premature aging syndromes and age-related neurological disease such as Parkinson’s disease (Diesch et al. 2014). Taken together, these observations show the importance of transcription and replication in the regulation and maintenance of rDNA repeat integrity. DNA damage can perturb these regulatory mechanisms and their interplay, thereby contributing to rDNA instability.

## Ribosomal DNA damage response

In response to DNA damage, cells halt cell cycle progression to allow for DNA repair. To avoid propagation of mutations, the DDR determines whether or not a cell continues to divide (Jackson and Bartek 2009). Different forms of DNA damage can be found but DSBs are among the most harmful and difficult lesions to repair (Hoeijmakers 2009). In response to DSBs, the two master regulators of the DDR, ataxia-telangiectasia mutated (ATM) and ataxia-telangiectasia and Rad3-related (ATR), become activated. ATM activation follows the accumulation of DNA ends, whereas ATR is recruited and subsequently activated after 5′–3′ resection of DSBs (Warmerdam and Kanaar 2010). ATM and ATR are kinases which phosphorylate many substrates, including histone H2AX (γH2AX), and play a pivotal role in the recruitment of numerous DDR-associated proteins to damaged sites. Perturbed transcription of rDNA has recently been shown to increase γH2AX levels, causing activation of p53, and correlating with neurological and developmental defects (Calo et al. 2018). In certain circumstances, the resolution of DSBs or intermediate DNA repair structures may remain unresolved, resulting in a persistent DDR, which is observed in senescent cells and aging organisms (Flach et al. 2014; Rodier et al. 2009; Kobayashi 2014; Noda et al. 2012). Unrepaired lesions may lead to error-prone repair resulting in mutations and chromosomal rearrangements (Noda et al. 2012). Notably, persistent breaks are frequently found to be associated with repetitive DNA sequences, like telomeres (Fumagalli et al. 2012) and may thus also be frequent in rDNA (Warmerdam et al. 2016). Altogether, the prevention, identification, and efficient resolution of DSBs, especially in repetitive DNA sequences, are of paramount importance for the maintenance of a cell’s genomic integrity and physiological function.

### Pathways involved in the repair of ribosomal DNA repeats

DSBs are predominantly repaired through direct ligation of the broken DNA ends by non-homologous end joining (NHEJ) or through HR (Kanaar et al. 2008; Ciccia and Elledge 2010). Because HR requires a homologous repair template, which is normally only present on the sister chromatid in S/G2 phase, the choice of DNA repair mechanism is regulated in the cell cycle (Shrivastav et al. 2008). Breaks in G1 are mainly repaired through NHEJ. In response to damage in the rDNA, rDNA transcription is shut down by the ATM-dependent inhibition of PolI (Harding et al. 2015). Next, rDNA DSBs relocate to the periphery of the nucleolus and form nucleolar caps (Harding et al. 2015; van Sluis and McStay 2015). To study repair of breaks in the 47S repeat, several labs have recently delivered I-PpoI into cells. I-PpoI is a sequence-specific endonuclease that cuts ~ 30 different locations in the human genome, including the 47S repeat (Warmerdam et al. 2016; Harding et al. 2015; van Sluis and McStay 2015). Harding et al. showed that breaks in rDNA repeats are predominately repaired through NHEJ (Harding et al. 2015). However, utilizing I-PpoI and CRISPR/Cas9 gene editing to induce DSBs specifically in rDNA, van Sluis & McStay concluded that rDNA breaks can also be repaired by HR, even in G1 cells (van Sluis and McStay 2015). They observed that HR-associated proteins also localize to rDNA break-induced nucleolar caps in G1 and that these repair structures show unscheduled DNA synthesis, a measure for ongoing repair. Importantly, the nucleolar caps contain both damaged and undamaged rDNA repeats, making templates available for HR regardless of the cell cycle. This interesting observation could have serious implications for human disease, and therefore, it will be important to investigate the role of HR-mediated repair of rDNA breaks in non-dividing cells in vivo, too.

In contrast, Warmerdam et al. showed that repair of rDNA breaks became more efficient after the loss of HR and that rDNA instability was dependent on homology-directed repair, indicating that recombination-mediated repair of breaks in the rDNA can result in a loss of repeat integrity (Warmerdam et al. 2016). Using CRISPR/Cas9 to investigate breaks in both the 5S and 47S repeats revealed that breaks in the 47S rDNA locus were more persistent and induced a stronger G2 checkpoint arrest than breaks in the 5S rDNA. The latter suggests that 47S breaks are more difficult to repair, maybe because the 47S rDNA is distributed over multiple chromosomes, unlike the 5S repeat, thereby creating more problems for homology-directed repair pathways. However, it is also important to note that the 47S rDNA is associated with the nucleoli, while 5S rDNA is not. This may indicate that spatial distribution of rDNA sites and pathways involved also influence repair of damaged rDNA repeats.

Warmerdam et al. have previously proposed that the observed HR-mediated loss of repeats after breaks in the rDNA occurs in *trans*, through recombination between sister chromatids or rDNA repeats on different chromosomes (Warmerdam et al. 2016). Unlike in S/G2, homology-dependent repair in G1/G0 might be more prone to occur in *cis*, by using unrepaired repeats on the same tandem array (van Sluis and McStay 2017). In addition, different homology-dependent repair mechanisms might be activated in either G1 and S/G2 cells (Renkawitz et al. 2014). Homology-dependent repair is subdivided in a number of pathways, which can mediate either *cis* or *trans*-dependent repair. Double-stranded break repair (DSBR) is the classical recombination pathway leading to chromosome crossovers (gene conversion) (Haber 2018). Synthesis-dependent strand annealing (SDSA) on the other hand suppresses crossovers and thereby prevents loss of heterozygosity (Verma and Greenberg 2016). As SDSA is mediated by BLM, the gene affected in Bloom syndrome, and loss of BLM results in rDNA instability, one can speculate that rDNA breaks are preferably repaired through this pathway, also to prevent loss of rDNA repeats.

**Fig. 2:**
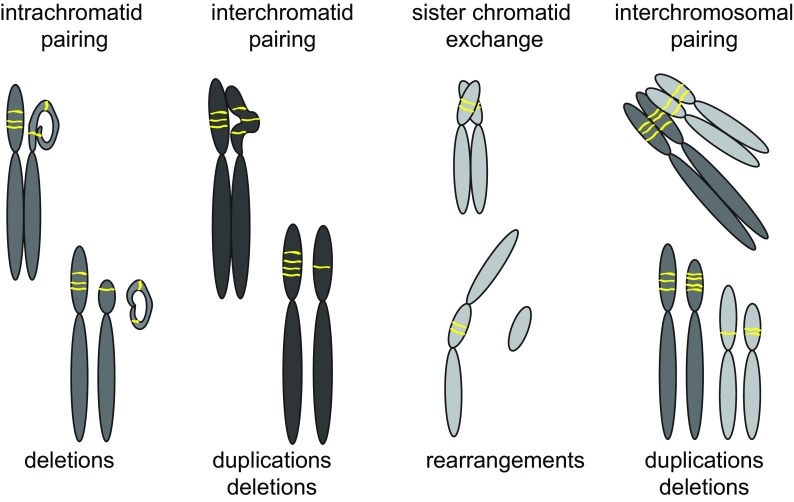
Repair of repeats can result in repeat expansions, contractions, and structural chromosomal aberrations. Configurations between chromosomes with repeats that can cause structural chromosomal rearrangements

Single-strand annealing (SSA) preferentially operates on short repetitive DNA sequence like CAG repeats. However, it is important to notice that comparing the repair of rDNA repeats to short repeats like telomeres and CAG repeats is troublesome as the rDNA is made up of very long repetitive sequences and therefore can result in different outcomes. SSA uses the homologous repetitive sequence adjacent to the damaged repeat for repair, and therefore does not result in crossovers. As such, repair through SSA is mutagenic and results in repeat contractions. Microhomology-mediated end joining (MMEJ), a process operating as an alternative to end joining, makes use of homology by using the bases directly adjacent to the breaks site as a repair template. This pathway is highly mutagenic and is involved in the alternative lengthening of telomeres (ALT) in cancer cell lines (Dilley and Greenberg 2015). SDSA, SSA, and MMEJ are expected to act in *cis* and therefore can result in intra-chromosomal repeat contractions (Fig. 2). Repair through DSBR works in *trans*, which could result in inter-chromosomal repeat expansions and contractions, but may also lead to structural chromosomal rearrangements (Fig. 2). However, it remains unclear whether dedicated homology-directed repair mechanisms operate on breaks in the rDNA and what the exact consequences of such repair mechanisms are for rDNA repeat integrity and genome stability.

### Processing of ribosomal DNA breaks and repair intermediates

DSB repair pathways often require processing of DNA ends and resolution of complex intermediates to generate suitable substrates for repair. BLM is a structure-specific helicase that plays a role in resolving rDNA structures (Killen et al. 2009). BLM is a highly conserved member of the RecQ family of helicases, important during recombination by promoting branch migration and resolving Holiday junctions (Karow et al. 2000; Cheok et al. 2005). However, BLM also suppresses recombination by disrupting Rad51 filament formation, a key process in HR, and promotes non-crossover recombination through SDSA (West et al. 2015). As such, BLM limits error-prone recombination between chromosomes (Wechsler et al. 2011). The WRN helicase, mutated in Werner Syndrome, is another RecQ helicase that promotes repair (Croteau et al. 2014). BLM and WRN both localize within the nucleolus and were shown to be involved in the regulation of PolI transcription (Tangeman et al. 2016; Shiratori et al. 2002; Grierson et al. 2012). WRN was also shown to be involved in maintaining rDNA stability (Caburet et al. 2005). However, loss of BLM leads to a more severe increase in rDNA instability compared to WRN loss (Killen et al. 2009), indicating that rDNA break repair is preferentially dependent on BLM.

The SMC5/6 complex, a highly conserved cohesion-like complex that is also responsible for sister chromatid interaction during HR, recruits BLM to resolve rDNA repair intermediates, but could also act as a platform for the recruitment of other structure-specific helicases and nucleases, possibly through SLX4 (Bermudez-Lopez and Aragon 2017). Repair of rDNA in yeast is mediated by SLX4, which functions as a scaffold for the recruitment of various nucleases (Coulon et al. 2006). In human cells, SLX4 is involved in the recruitment of nucleases including ERCC1, Mus81, and SLX1 to sites of damage (Munoz et al. 2009). It remains unclear whether structure-specific nucleases also play a role in the processing of rDNA repair intermediates in human cells. Mass-spectrometry of the nucleolar proteome also indicates the presence of the flap endonuclease FEN1 (Andersen et al. 2002). However, nucleases could also be recruited to nucleolar caps in response to rDNA breaks. It seems reasonable that repair-dependent helicases and nucleases will eventually need to access unresolved rDNA repair intermediates in order to prevent persistent breaks. Future investigations will undoubtedly show whether processing of rDNA during repair is important for the maintenance of rDNA integrity in human cells.

### Maintaining ribosomal DNA repeat integrity during repair

Cohesin is a highly conserved protein complex that mediates the cohesion between sister chromatids upon their replication and regulates their timely separation during mitosis (Peters et al. 2008; Peters and Nishiyama 2012). Sister chromatid cohesion facilitates rRNA synthesis by an unknown mechanism. It has been suggested that cohesin stabilizes a looping structure that facilitates reloading of PolI, or that it promotes replication fork speed (Lu et al. 2014). Yeast cells containing reduced numbers of rDNA repeats show enhanced rDNA transcription on the remaining repeats. Interestingly, a presumed inability of cohesin to bind to transcriptionally active repeats is associated with improper sister chromatid alignments, error-prone recombination, and rDNA instability (Ide et al. 2010). This observation also implies that both silent and active repeats are required to regulate recombination and maintain rDNA integrity.

In yeast, the SMC5/6 protein complex, a highly conserved cohesion-like complex that is also responsible for sister chromatid interaction during HR, contributes to rDNA stability (Torres-Rosell et al. 2007; Bose et al. 2012; Lu et al. 2014). Mutants of this complex show increased formation of X-shaped DNA structures also known as Holliday junctions in the rDNA. These are alleviated by co-repression of the recombination-associated protein Rad51, indicating that rDNA instability in these mutants is caused by inappropriate HR. It has been proposed that SMC5/6 is highly concentrated within the nucleolus where it locates to DSBs in the rDNA and prevents the formation of Rad51, thus suppressing HR (Eckert-Boulet and Lisby 2009; Torres-Rosell et al. 2007). In response to rDNA breaks, SMC5/6 also acts as a platform for the SUMOylation of target proteins like the SSA-promoting enzyme Rad52, although the molecular mechanisms and interactions influenced by this post-translational modification are unclear (Potts 2009). In addition, the SMC5/6 complex recruits the DNA helicase BLM to resolve intermediate DNA structures during rDNA break repair, possibly through SDSA (Killen et al. 2009).

According to one proposed mechanism, SMC5/6 and factors recruited in response to rDNA DSBs serve to decrease recombination events between different rDNA repeats and thereby prevent instability when the rDNA is in the nucleolus. Damaged rDNA is then moved to the periphery of the nucleolus, locating to nuclear caps together with HR factors, where SMC5/6 is off-loaded and recombination allowed to proceed (Eckert-Boulet and Lisby 2009). This model also highlights the importance of spatial regulation of repair proteins in response to damaged rDNA repeats. Although SMC5 has recently been implicated in the repair of rDNA breaks in human cells (Warmerdam et al. 2016), most of what we know about the regulation of rDNA stability comes from other model organisms. Further studies using human cells should indicate whether the identified mechanisms and proposed models are conserved.

## Human disease and the mechanisms that maintain rDNA integrity

Taken together, maintenance of rDNA stability and control of transcriptional output of rRNA are highly regulated processes that we are just beginning to understand. It is also becoming clear that cells have evolved multiple intertwined processes to regulate ribosome biogenesis, also to adapt to changing conditions. By accommodating alterations in rDNA repeat length and transcriptional output, cells would be able to quickly adjust protein synthesis rates. For example, the regulation of repair pathway choice after breaks in the rDNA repeats through processing and cohesion-dependent structural alignment will enable cells to influence the stability of the rDNA as well as its transcription. We expect that the spatial distribution of the nucleolus and factors that maintain rDNA integrity, which we did not discuss in detail here, could also play an important role in controlling rDNA stability. These systems can however also be subject to mistakes, and deregulation can lead to hereditary disorders and human diseases that possibly involve neurodegeneration and tumorigenesis.

Whereas we have learned a lot about the DDR in recent years, it remains to be discovered how discrete breaks in repetitive sequences are dealt with. Repair of repetitive DNA, including the rDNA repeats, is differently regulated compared to non-repetitive DNA sequences. Understanding this process and its potential impact on genome stability and disease requires dedicated studies of, for instance, the DNA sequence in which such breaks occur. The development of CRISPR/Cas and other new methods will enable these investigations.

## Methodological advancements

**Fig. 3:**
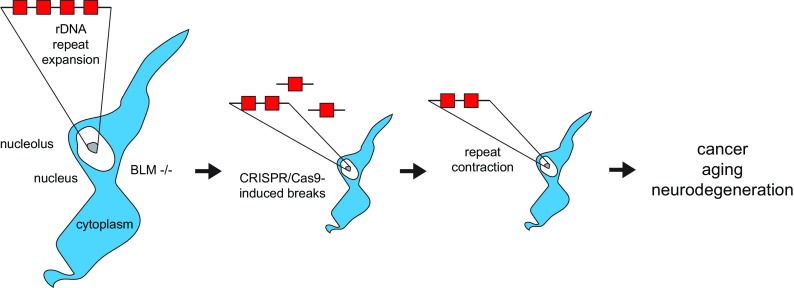
Reversing repeat instability through CRISPR/Cas9. Can a reduction in the number of rDNA repeats in BLM deficient cells by CRISPR/Cas9-induced breaks rescue clinically relevant features including neurodegeneration, cancer, and aging?

In order to uncover new layers of genome maintenance that are relevant for human disease, we will need to overcome some of the difficulties in studying rDNA repeats. Investigating repeat copy numbers using sequencing approaches is problematic as the number of repetitive DNA sequences is not well annotated and repeat amplification by PCR can be biased. The quantification of rDNA copy numbers using digital droplet PCR forms an alternative approach to overcome this hurdle (Salim et al. 2017; Xu et al. 2017a, b). Using transcription activator-like effector nuclease (TALEN), it was possible to visualize rDNA repeats in individual cells. By analyzing rDNA copy numbers using fluorescence intensity in single cells, researchers observed a reduction of rDNA repeats during aging (Ren et al. 2017). Similar to fluorescent in situ hybridization (FISH), visualization using a fluorescently labeled version of inactive Cas9 (dCas9) will enable the quantification of repeats in individual cells and possibly per chromosome. Since, unlike FISH, CRISPR/dCas9 fluorescent labeling of genes is compatible with living cells, this approach would allow the study of the behavior of rDNA repeats after breaks. The dCas9 system can also be used to modulate rDNA transcription by using a dCas9 coupled to either the transcriptional inhibitor KRAB or activator VP64. Additionally, epigenetic regulators like histone (de)acetyl transferases could be used to study the effects of re-activing silenced repeats or repressing them. Interestingly, epigenome editing using CRISPR/dCas9 was recently used to silence microsatellite repeats involved in tumorigenesis (Boulay et al. 2018), showing that repetitive DNA can be a tumor-specific vulnerability that might be exploitable in cancer therapy. Moreover, various CRISPR-mediated approaches can be combined by applying different versions of Cas9 that recognize a range of different PAM sequences (Cong and Zhang 2015), increasing the possibilities to generate gene-specific markers. CRISPR-approaches have already been successfully used to edit deleterious CAG repeat extensions and similar approaches might be possible to target cells with altered rDNA repeats (Massey and Jones 2018; Dabrowska et al. 2018; Cinesi et al. 2016) (Fig. 3).

## Clinical advancements

Interestingly, alterations in rDNA copy numbers may predict therapy responses (Wang and Lemos 2017; Xu et al. 2017a, b). This phenomenon might be further exploited in cancer therapy, for instance by using specific inhibitors of ribosome biogenesis, as recently been shown in ATRX-mutated ALT-positive cancers (Udugama et al. 2018). Selective PolI inhibitors block rDNA transcription, disrupt nucleolar function, and were shown to selectively kill tumor cells in vivo while sparing normal cells, presumably as a result of impaired ribosome biogenesis (Hannan et al. 2013a, b; Hein et al. 2013). Increased ribosome activity in cancer might form an Achilles’ heel, permitting selective targeting of tumor cells by PolI inhibitors (Sluis and McStay 2014). Although we are beginning to understand more about connections between ribosome biogenesis and tumorigenesis, there are no biomarkers available yet to predict which types of tumors would be susceptible to rDNA transcription or other forms of interference with ribosome biogenesis. Several chemical compounds have shown to inhibit PolI activity: Actinomycin D is a naturally occurring polypeptide antibiotic that intercalates at GC-rich regions in the DNA and thereby inhibits PolI, but it also inhibits the activity of the other two RNA polymerases (II and III) and is highly toxic. Two recently described drugs, CX-5461 and BMH-21, are reported to inhibit PolI activity more specifically, with promising therapeutic potential in the treatment of cancer. CX-5461 was found to inhibit PolI transcription by disrupting pre-initiation complex formation at the rDNA promoter (Bywater et al. 2012). This induced a p53-dependent and p53-independent signaling response without inducing DNA damage, leading to selective cell death in cancer cells while normal cells were largely unaffected (Quin et al. 2014). However, it was also reported recently that CX-5461 stabilizes G-quadruplexes (Xu et al. 2017a, b). In this study, exposure to CX-5461 blocked replication forks and resulted in DNA damage. Recombination-deficient cancer cells were highly sensitive to CX-5461, since DNA repair was required to deal with CX-5461-induced damages. An alternative explanation for these results is that repair-deficient cells already accumulate G4 structures in the rDNA during replication, which block rDNA transcription, leading to R-loops and eventually DSBs. Combined, these results imply that CX-5461 might affect cancer cells through different mechanisms. BMH-21 inhibits PolI activity by promoting the degradation of the PolI catalytic subunit RPA194 (Peltonen et al. 2014b), resulting in checkpoint activation and cell death without the occurrence of a strong DNA damage response (Colis et al. 2014; Peltonen et al. 2014a). Interestingly, Oxaliplatin, a crosslinking agent and widely used chemotherapeutic, has recently been reported to sensitize tumor cells by inhibiting ribosome biogenesis, too (Bruno et al. 2017). Although dependent on further development of specific inhibitors, clearly, inhibition of ribosome biogenesis is emerging as a potential target in cancer therapy.

Oncogenic transformation is often linked to altered PolI activity. The MYC family of transcription factors belongs to the most pervasive oncogenes and activation of MYC correlates with poor prognosis (Dang 2012). MYC recognizes target gene promoters by direct DNA binding, but can also be recruited through indirect protein–protein interactions with other transcription factors. Oncogenic MYC has shown to enhance PolI transcription, leading to enhanced ribosome biogenesis (Devlin et al. 2016; Poortinga et al. 2015; Drygin et al. 2011; Quin et al. 2014; Hein et al. 2013). The addiction of MYC-driven cancers to enhanced ribosome activity has emerged as a vulnerability which might be exploited in cancer therapy (Sluis and McStay 2014; Ruggero 2012). Another link to cancer relates to the PI3K pathway which is frequently activated, e.g., by loss of the tumor suppressor PTEN. PI3K supports rDNA transcription by enabling PolI association to the rDNA promoter (Zhang et al. 2005; Kusnadi et al. 2015). Recently, a strictly nucleolar PTEN isoform (PTENβ) was identified as the negative regulator of rDNA transcription (Liang et al. 2017). This suggests a mechanism by which PTENβ deficient tumors increase ribosome biogenesis and cellular proliferation, which may also contribute to ribosome addiction in such tumors (Dillon and Miller 2014). Altogether, a better understanding of the connection between the regulation of rDNA through copy number and transcription and ribosome biogenesis in cancer may lead to the identification of additional biomarkers to predict sensitivity towards PolI inhibitors or other ways to interfere with aberrant ribosome biogenesis.

## Perspective

It is becoming evident that rDNA repeat stability plays an important role in human disease. Cells are especially vulnerable to damage in the rDNA, due to intrinsic risks for error-prone repair, leading to rDNA copy number alterations or structural chromosomal aberrations. To further elucidate the role of rDNA integrity in genome stability and disease, we will need to answer the following key questions: (i) how do human cells regulate the integrity of rDNA repeats, (ii) can break-induced rDNA repeat alterations instigate genomic instability, and (iii) is altered repeat instability is a significant inducer of ribosome biogenesis, and thereby a druggable cancer vulnerability? A better understanding of the molecular pathways that involved rDNA repeat maintenance is needed to clarify these matters. As a first step, identification of proteins protecting repeat stability in the presence of DNA damage could reveal the molecular connections between rDNA repeats, genome integrity, and ribosome biogenesis. This will help to predict the importance of rDNA stability in preventing disease and may point towards targeted strategies for therapeutic intervention. Furthermore, we expect that CRISPR/Cas-related tools will aid in following dynamic alterations in rDNA repeat lengths, and create tools to interfere with rDNA integrity to test its role in ribosome biogenesis and disease-related alterations in protein synthesis. These will help to uncover the molecular mechanisms governing rDNA repeat stability, supporting the identification of novel disease biomarkers and provide new strategies towards the development of tailored treatment options.

## Abbreviations

rDNA: Ribosomal DNA
rRNA: Ribosomal RNA
mRNA: Messenger RNA
DSBs: DNA double-stranded breaks
ALS: Amyotrophic lateral sclerosis
FTD: Frontotemporal dementia
DDR: DNA damage response
Fob1: Fork blocking protein 1
RFB: Replication fork blocking
PolI: RNA polymerase I
HR: Homologous recombination
PHF6: Plant homeodomain finger protein 6
NuRD: Nucleosome remodeling deacetylase
NoRC: Nucleolar chromatin remodeling complex
ATM: Ataxia-telangiectasia mutated
ATR: Ataxia-telangiectasia and Rad3-related
γH2AX: Phosphorylated histone H2AX
NHEJ: Non-homologous end-joining
DSBR: Double-stranded break repair
SDSA: Synthesis-dependent strand annealing
SSA: Single-strand annealing
MMEJ: Micro homology-mediated end-joining
ALT: Alternative lengthening of telomeres
TALEN: Transcription activator-like effector nuclease
FISH: Fluorescent in situ hybridization
